# Supplementary material for: The suboptimality of perceptual decision making with multiple alternatives
Source: Nat Commun. 2020 Jul 31;11:3857. doi: 10.1038/s41467-020-17661-z (PMC7395091; doi:10.1038/s41467-020-17661-z)
Supplement: Supplementary file 1 — Supplementary Information [file 41467_2020_17661_MOESM1_ESM.docx]

**SUPPLEMENTARY INFORMATION**

**The suboptimality of perceptual decision making with multiple alternatives**

Jiwon Yeon and Dobromir Rahnev

**Supplementary Methods**

Mathematically deriving the difference in accuracy between the population and summary models in the 2-alternative condition

In the Results section on Experiment 1, we described the intuition regarding why the population model predicts higher accuracy than the summary model in the 2-alternative condition (Figure 3). Here we provide a precise mathematical formulation. Note that the derivations below assume the absence of a lapse rate, which would act to attenuate but not remove the differences between the accuracy levels predicted by the two models.

Let $p_{dom=i}$ be the probability that, for a particular set of parameters describing the sensory response, the dominant stimulus produces the $i^{th}$ highest activation. Then, the accuracy in the 4-alternative condition would simply equal $p_{dom=1}$ as correct trials require that the dominant color produces the highest activation. We can then derive the accuracy in the 2-alternative condition for the population and summary models.

To compute the accuracy in the 2-alternative condition for the population model, we can derive the expected accuracy when the dominant stimulus produces the $i^{th}$ highest activation for $i=1,2,3,4.$ When $i=1$, the dominant stimulus produces the highest activation and the subject is always correct. When $i=2$, the subject is correct when the alternative option happened to be the stimulus with 3^rd^ or 4^th^ highest activation but is wrong when the alternative option happened to be the stimulus with the highest activation. Because the alternative option was chosen randomly, the probability of being correct in this case is $\frac{2}{3}$. For similar reasons, when $i=3$, the probability of being correct is $\frac{1}{3}$. Finally, when $i=4$, the subject would be wrong regardless which non-dominant stimulus is chosen as the alternative option. Therefore, $Acc_{pop-model,2-alt}$, the overall accuracy of the population model in the 2-alternative condition, is:

$Acc_{pop-model,2-alt}=p_{dom=1}+\frac{2}{3}*p_{dom=2}+\frac{1}{3}*p_{dom=3}$ (1)

To compute the accuracy in the 2-alternative condition for the summary model, we can again derive the expected accuracy when the dominant stimulus produces the $i^{th}$ highest activation for $i=1,2,3,4.$ As with the population model, when $i=1$, the dominant stimulus produces the highest activation and the subject is always correct. However, unlike the population model, $i=2,3,4$ produce the same probability of being correct. Indeed, in all of these cases, the dominant stimulus does not produce the highest activation and the summary model does not have access to the activations other than the highest activation. From the remaining three stimuli, there is a $\frac{1}{3}$ chance that the stimulus with the highest activation was chosen as one of the two options, in which case the subject is always wrong. On the other hand, with $\frac{2}{3}$ chance another stimulus that did not produce the highest activation is chosen as the alternative option. Because the subject chooses randomly in this case, the probability of being correct is $\frac{1}{2}$. Therefore, the probability of being correct for $i=2,3,4$ is always $\frac{2}{3}*\frac{1}{2}=\frac{1}{3}$ and $Acc_{summary-model,2-alt}$, the overall accuracy of the summary model in the 2-alternative condition, is:

$Acc_{summary-model,2-alt}=p_{dom=1}+\frac{1}{3}p_{dom=2}+\frac{1}{3}*p_{dom=3}+\frac{1}{3}*p_{dom=4}$ (2)

From here we obtain that the difference between the accuracy of the population and summary models in the 2-alternative condition is $\frac{1}{3}\left( p_{dom=2}-p_{dom=4} \right)$. Because the dominant stimulus is at least as likely to produce the second highest than the 4^th^ highest activation, $p_{dom=2}-p_{dom=4}\geq0$, which means that the population model predicts higher accuracy in the 2-alternative condition compared to the summary model. Similar derivations can be made for Experiments 2-4 as well.

Analytical expression for model behavior

For completeness, we provide formulas for $p_{ij}$, the predicted probability of giving a response $i$ when stimulus $j$ is presented. Note that as in the section above, these expressions assume the absence of a lapse rate. When initially fitting the model to the 4-alternative condition in Experiment 1, $p_{1j}$ equals:

$$p_{1j}=P\left( x{}_{1j}>\max\left( x_{2j},x_{3j},x_{4j} \right) \right)=$$

$$=\int_{-\infty}^{\infty} \int_{-\infty}^{x{}_{1j}} \int_{-\infty}^{x{}_{1j}} \int_{-\infty}^{x{}_{1j}} f\left( x{}_{1j} \right| \mu_{1j},1)f\left( x{}_{2j} \right| \mu_{2j},1)f\left( x{}_{3j} \right| \mu_{3j},1)f\left( x{}_{4j} \right| \mu_{4j},1) dx{}_{4j} dx{}_{3j} dx{}_{2j} dx{}_{1j}$$

(3)

where $\mu_{ij}$ is the mean activity for option $i$ when stimulus $j$ is presented, $x{}_{ij}$ is the activity on a specific trial for option $i$ when stimulus $j$ is presented, and $f\left( x \right| \mu,1)= \frac{1}{\sqrt{2\pi}} e^{-\frac{\left( x-\mu\right)^{2}}{2}}$ is the Gaussian probability distribution of sensory evidence. The probability $p_{ij}$ can be computed in an equivalent fashion when $i\neq1$ and when the total number of stimulus categories is different than four (as in Experiments 2-4). Similar formulas can be obtained when fitting the summary and population models to the 2-alternative condition.

Model development for all additional models

In addition to the population and summary models, we considered four other models. These models were developed in order to test additional hypotheses about the nature of the representation at the decision stage and the strategies that our subjects could have used. We have not extended this set of four more models even further because any additional models were judged to be too ad hoc and generally provided even worse fits to the data.

The first two of the additional models postulated that decision-making circuits contain information about the sensory representation that is more detailed than the summary model but less detailed than the population model. Specifically, we created models according to which decision-making circuits have access to the highest two or three levels of activation (2-Highest and 3-Highest models, respectively). Just as the summary and population models, these two models were used to predict task performance in the 2-alternative condition of Experiments 1-2 without any free parameters (the predictions were derived from the same model of the sensory representation used for the summary and population models). Note that the 3-Highest model is functionally equivalent to the population model in the context of Experiment 1 and both the 2- and 3-Highest models are functionally equivalent to the population model in the context of Experiments 3 and 4.

The last two models postulated that subjects attended to just two or three stimulus categories (i.e., colors or symbols) on each trial and made their decisions based on a full probability distribution over the activity levels of the attended categories. We called these the 2-Attention and 3-Attention models, respectively. The intuition behind these models is that subjects may not be able to process well the whole set of categories and may therefore choose to focus only on a subset of the categories. The subset was chosen randomly on each trial (otherwise, if subjects always ignored a given stimulus category, that category will never be selected; however, we never observed such behavior in any of our subjects). We first fit these models to the 4-alternative condition (Experiment 1), 6-alternative condition (Experiment 2), and the first answer (Experiment 3) in order to create a model of the sensory representation. The models were then used to predict the 2-alternative condition (Experiments 1 and 2) or the second answer (Experiment 3) without any free parameters. This procedure was equivalent to the procedure used for the summary and population models.

**Supplementary Notes**

Besides the population and summary models, we constructed and tested four additional models. The 2-Highest and 3-Highest models postulated that decision-making circuits have access to the two or three highest activations of the sensory distribution, respectively. These models thus assume a less severe loss of information compared to the summary model while still postulating that the whole sensory code is not represented in decision-making circuits. On the other hand, the 2-Attention and 3-Attention models postulate that subjects choose either two or three stimulus categories to attend to and then make their decisions based on a full probability distribution over the activity levels of the attended categories. We found that neither of these models outperformed the summary model in any of our experiments.

Experiment 1

The 2-Highest model (average predicted accuracy = 83.5%) significantly overestimated the observed accuracy level for the 2-alternative condition (average difference = 5.46%; *t*(31) = 7.49, *p* = 1.94 x 10^-8^, 95% CI = [4%, 7%]; Supplementary Figure 1a). Moreover, the absolute errors in the prediction of the 2-Highest model for the 2-alternative condition (average = 5.46%) is larger compared to the summary model (*t*(31) = 4.78, *p* = 4.07 x 10^-5^, 95% CI = [1.2%, 3.2%]). Model comparison favored the summary model over the 2-Highest model by an average 11.86 AIC points (corresponding to the summary model being 375.41 times more likely for the average subject) and by 379.39 AIC points in the group as a whole (corresponding to the summary model being 2.42 x 10^82^ times more likely in the group; Supplementary Figure 1b and c). Note that within the context of Experiment 1, the 3-Highest model is functionally equivalent to the population model. Indeed, according to the 3-Highest model, the activity level that is not represented is always the lowest; therefore, the 3-Highest model allows one to still order all four activity levels in descending order making it equivalent to the population model.

The 2- and 3-Attention performed even worse. These models could not even be fit to the data in the 4-alternative condition with both models predicting much lower performance (2-Attention model: average difference = -23.8%, *t*(31) = 22.5, *p* = 9.08 x 10^-21^, 95% CI = [-26%, -22%]; 3-Attention model: average difference = -6.73%, *t*(31) = 10.68, *p* = 6.47 x 10^-12^, 95% CI = [-8%, -5%]; Supplementary Figure 6a). Nevertheless, we still generated the predictions of these models for the 2-alternative condition and again found that they strongly underpredicted the observed accuracy (2-Attention model: average difference = -21.2%, *t*(31) = 24.84, *p* = 5 x 10^-22^, 95% CI = [-23%, -20%]; 3-Attention model: average difference = -8.36%, *t*(31) = 11.18, *p* = 2.08 x 10^-12^, 95% CI = [-10%, -7%]; Supplementary Figure 6b,c). Finally, when compared to the summary model, both models showed much worse fit (2-Attention model: average AIC difference = 1.19 x 10^14^; 3-Attention model: average AIC difference = 8.12 x 10^3^; Supplementary Figure 6d).

Experiment 2

The 2-Highest model overestimated the observed accuracy in the 2-alternative condition (74.9%; *t*(9) = 5.65, *p* = 3.12 x 10^-4^, 95% CI = [2%, 5%]) and provided worse fit to the data compared to the summary model (average AIC difference = 18.81 points, total AIC difference = 188.07 points; Supplementary Figure 2). The 3-Highest model fared even worse. It overestimated the accuracy in the 2-alternative condition even more severely (76.7%; *t*(9) = 8.27, *p* = 1.69 x 10^-5^, 95% CI = [4%, 6%]) and provided much worse fit to the data compared to the summary model (average AIC difference = 39.87 points, total AIC difference = 398.65 points; Supplementary Figure 2).

Similar to the result in Experiment 1, the 2- and 3-Attention models could not even be fit to the 6-alternative condition with both models predicting much lower performance (2-Attention model: average difference = -21.6%, *t*(9) = -8.07, *p* = 2.07 x 10^-5^, 95% CI = [-28%, -16%]; 3-Attention model: average difference = -11.3%, *t*(9) = -5.50, *p* = 3.80 x 10^-4^, 95% CI = [-16%, -7%]; Supplementary Figure 6e). We still generated the predictions of these models for the 2-alternative condition and again found that they strongly underpredicted the observed accuracy (2-Attention model: average difference = -19.2%, *t*(9) = -10.25, *p* = 2.91 x 10^-6^, 95% CI = [-23%, -15%]; 3-Attention model: average difference = -14.2%, *t*(9) = -8.35, *p* = 1.57 x 10^-5^, 95% CI = [-18%, -10%]; Supplementary Figure 6f,g). Finally, when compared to the summary model, both models showed much worse fit (2-Attention model: average AIC difference = 2.71 x 10^61^; 3-Attention model: average AIC difference = 1.30 x 10^37^; Supplementary Figure 6h).

Experiment 3

The design of Experiment 3 made the 2- and 3-Highest models functionally equivalent to the population model (and thus their predictions were equivalent to that model). Similar to Experiments 1 and 2, the 2- and 3-Attention models did not fit well to the first answer (2-Attention model: average difference = -22%, *t*(9) = -8.41, *p* = 1.48 x 10^-5^, 95% CI = [-28%, -16%]; 3-Attention model: average difference = -11.2%, *t*(9) = -5.10, *p* = 6.47 x 10^-4^, 95% CI = [-16%, -6%]; Supplementary Figure 6i). We still generated the predictions of these models for the second answer and again found that they strongly underpredicted the observed accuracy (2-Attention model: average difference = -23.1%, *t*(9) = -12.87, *p* = 4.23 x 10^-7^, 95% CI = [-27%, -19%]; 3-Attention model: average difference = -15.9%, *t*(9) = -7.46, *p* = 3.84 x 10^-5^, 95% CI = [-21%, -11%]; Supplementary Figure 6j,k). Finally, when compared to the summary model, both models showed much worse fit (2-Attention model: average AIC difference = 3 x 10^48^; 3-Attention model: average AIC difference = 3.09 x 10^14^; Supplementary Figure 6l).

**Supplemenary Figures**

­­
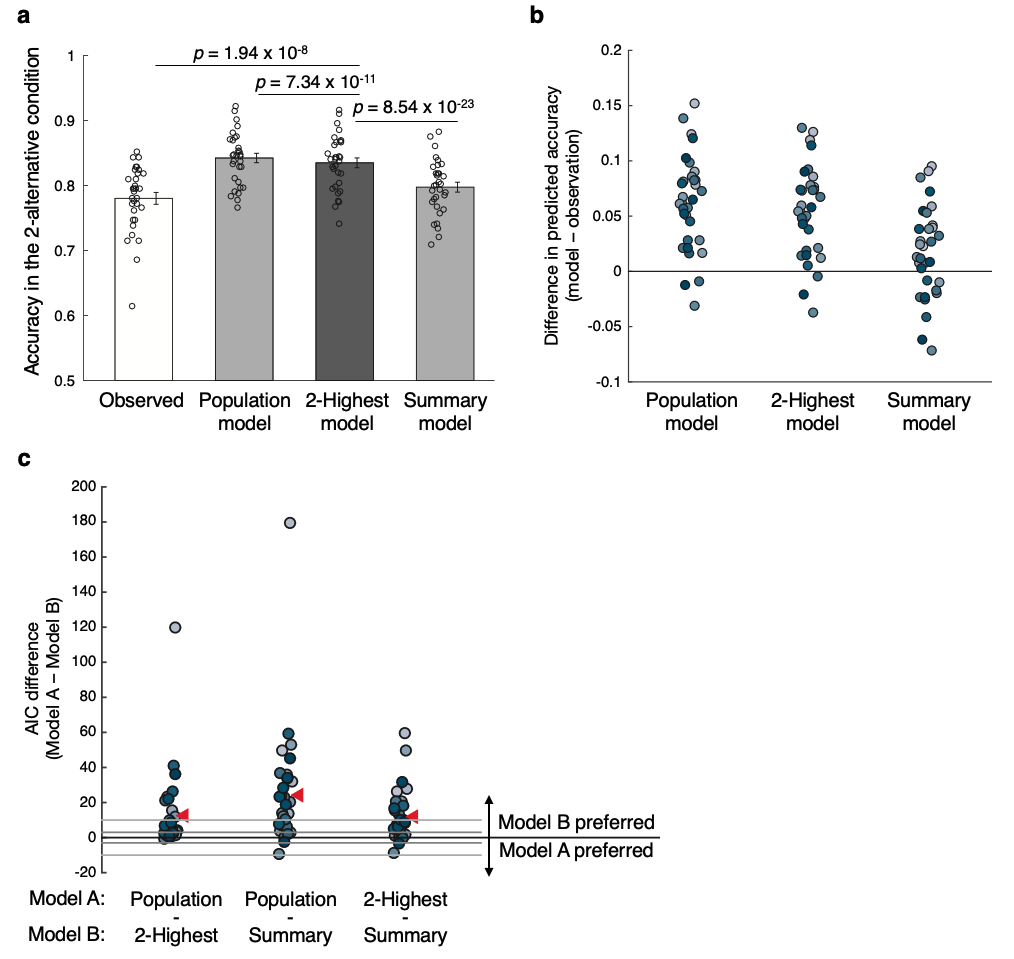


**Supplementary Figure 1. Results for the 2-Highest model in Experiment 1**. The results regarding the population and summary models are the same as in Figure 4. (a) Mean task accuracies of the actual data (white bar; 78% accuracy), the population and summary model (light gray bars) and the 2-Highest model (dark gray bar). The predicted accuracy of the 2-Highest model (83.5%) is in between the accuracy predicted by the population (84.2%) and summary (79.7%) models. All p-values are derived from two-sided paired t-tests. Error bars represents SEM, n = 32. (b) Difference in the accuracy for the 2-alterantive condition between the model predictions and the observed data. The deviations in predicted accuracy of the 2-Highest model are generally higher than for the summary model. (c) Difference in Akaike Information Criterion (AIC) between the three models. Positive AIC values indicate that the model subtracted (i.e., Model B) provides a better fit to the data. The 2-Highest model provides better fits than the population model, but worse than the summary model in the majority of the subjects.

­­
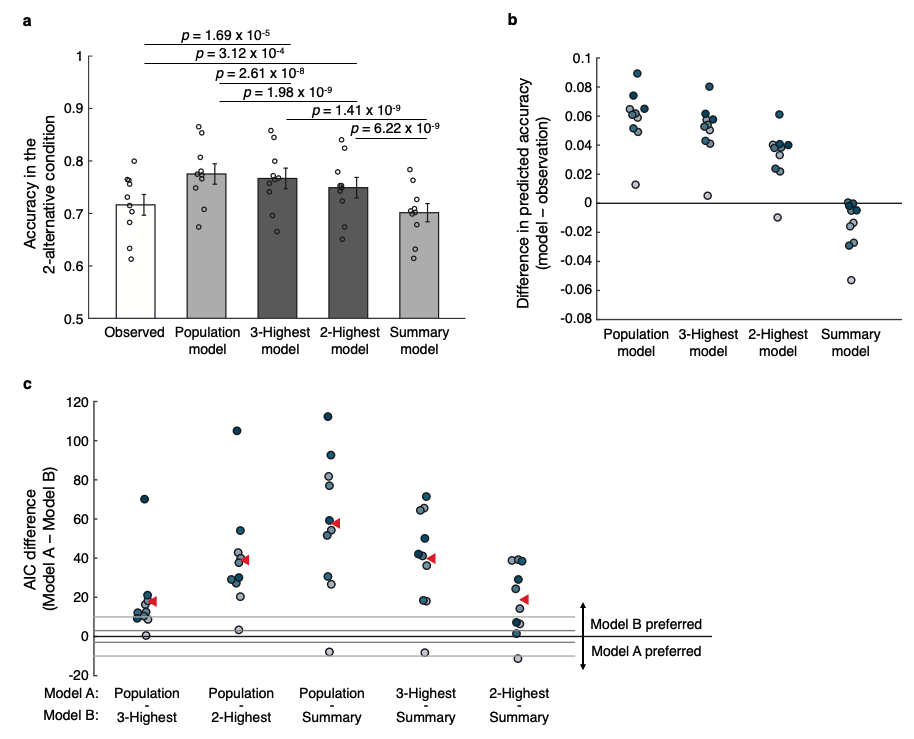


**Supplementary Figure 2. Results for the 2- and the 3-Highest models in Experiment 2**. The results regarding the population and summary models are the same as in Figure 5. (a) Mean task accuracies of the actual data (white; 71.6% accuracy) and the four models. Similar to the result in the Experiment 1, the predicted accuracies of the 2- and the 3-Highest model (74.9% and 76.7% respectively) fell in between the predicted accuracies of the population (77.5%) and the summary (70.1%) models. All p-values are derived from two-sided paired t-tests. Error bars represents SEM, n = 10. (b) Difference in task accuracy between the models and the observed data. (c) Model fit comparison between the three models. The summary model has the lowest AIC values, followed by the 2-Highest, the 3-Highest, and the population model.

_­_
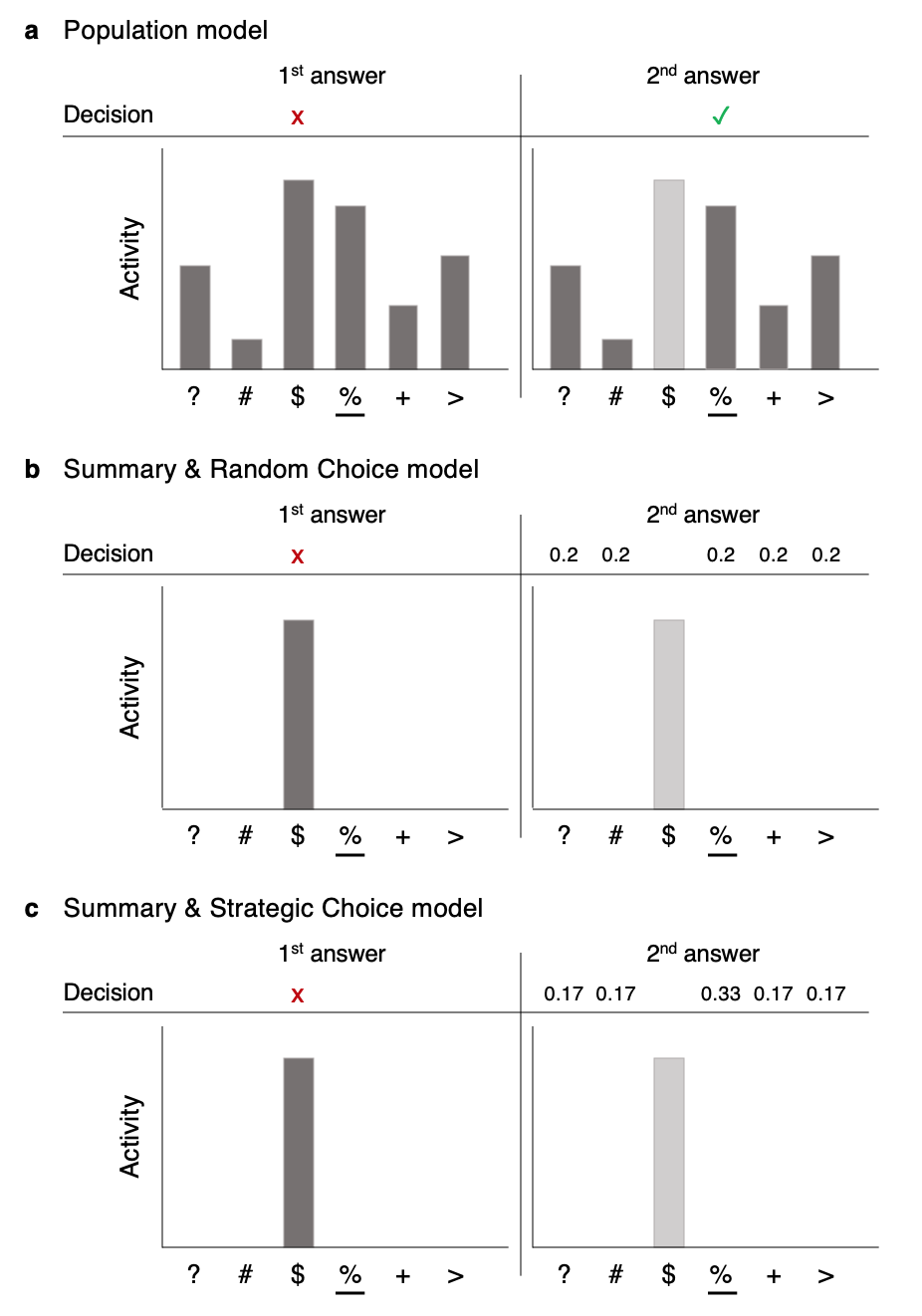


**Supplementary Figure 3. Predictions of each model for the second answer in Experiment 3**. In all examples, the percentage sign was the dominant symbol but the dollar sign produced the highest activity. The first answer is always the same across all models (left panels) – each model postulates that the highest activity will be chosen first. The predictions diverge for the second answer (right panels; the activity for the dollar sign is represented in a light gray bar to indicate that it cannot be chosen again). (a) According to the population model, decision-making circuits have access to the activity levels associated with all symbols (dark gray bars). Therefore, the population model would imply that the second answer will have a relatively high accuracy since the dominant symbol is likely to have higher activity than the other symbols. (b) According to the summary models, decision-making circuits do not have information about anything but the most highly activated symbol. After an incorrect response, according the Summary & Random Choice model, subjects pick an answer randomly, resulting in 20% accuracy level. (c) According to the Summary & Strategic Choice model, subjects choose the second answer strategically. Specifically, the model postulates that subjects choose the stimulus category of a randomly recalled symbol from the 7x7 grid (see Supplementary Figure 4 for details). Therefore, according to the Summary & Strategic Choice model, the accuracy for the second answer will be 33.3%.

**Supplementary Figure 4. Strategy assumed for the Summary & Strategic Choice model**. In the example above, a subject incorrectly chooses the dollar sign with their first answer (top panel; the correct answer is the percentage sign). The model assumes that for their second answer, subjects recollect a single symbol from the original display that was not their first choice and respond with it. Indeed, given that subjects inspected the stimuli for 500 ms, they could easily remember one location with a symbol other than the one they picked for their first answer. With this strategy, the probability that the second answer would be correct is 33.3%, since there were 14 instances of the dominant symbol and 42 locations in the grid (discounting the locations occupied by the symbol chosen with the first answer). Similarly, the probability of picking any specific wrong symbol as the second choice would be 16.7%, since there were 7 instances of that non-dominant symbol and 42 total remaining location in the grid.

**
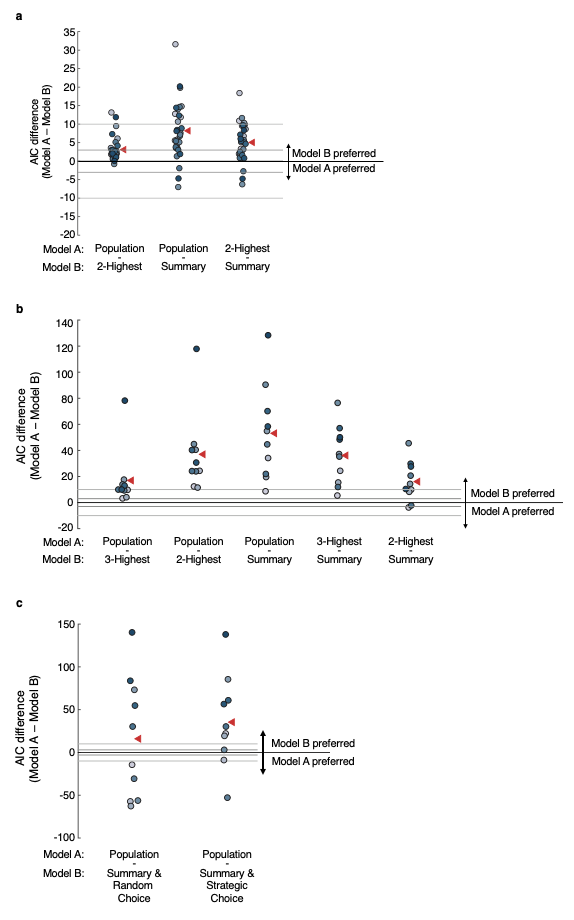
**

**Supplementary Figure 5. Results of an alternative way of modeling the sensory response**. In the analyses reported in the main paper, we modeled the activation levels of each stimulus category (i.e., colors in Experiment 1 and symbols in Experiments 2 and 3) differently depending on the identity of the dominant stimulus. The number of free parameters was thus 13 in Experiment 1 (4 possibilities for the dominant color $\times$ 3 free parameters to model the activation for each stimulus category + 1 lapse rate) and 31 in Experiments 2 and 3 (6 possibilities for the dominant symbol $\times$ 5 free parameters to model the activation for each stimulus category + 1 lapse rate). We re-analyzed our data using the simplifying assumption of independence between the activations produced by a given stimulus category and the identity of the dominant color. In other words, for example, the color green when non-dominant was assumed to produce the same average activation regardless of whether the dominant color was red, blue, or white. This simplifying assumption decreased the number of free parameters significantly: There were eight free parameters in Experiment 1. The free parameters were used to model the activations of 4 stimulus categories $\times$ 2 possible states (dominant/non-dominant) and an additional parameter was used for the lapse rate. However, because adding a constant to all activation parameters retains the relationship between them, one of these parameters was set as zero, bring the total number of free parameters to eight. Similarly, the six symbols in Experiments 2 and 3 resulted in 12 total free parameters. The figure shows model comparison results for this simplified modeling architecture for (a) Experiment 1, (b) Experiment 2 and (c) Experiment 3. In all cases, the summary model is preferred over the population model, typically to the same extent as in the main analyses. Thus, despite the differences between the simplified modeling architecture and the analyses reported in the main experiment, both led to essentially the same results.


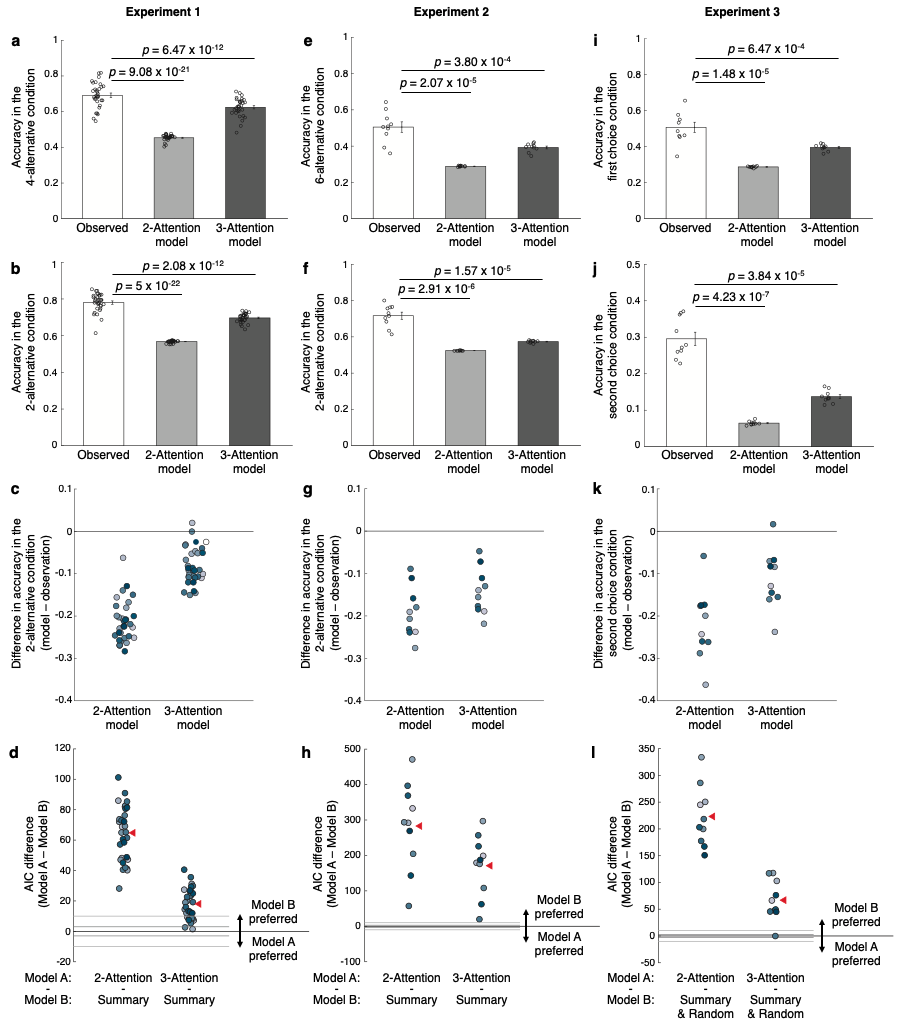


**Supplementary Figure 6. Results of the 2- and 3-Attention models**. (a, b, e, f, i, and j) Mean accuracy predicted by 2- (light gray bar) and 3-attention (dark gray bar) models and observed in the actual data (white bar) respectively in Experiment 1 (a and b; n = 32), 2 (e and f; n = 10), and 3 (i and j; n = 10). All p-values are derived from two-sided paired t-tests. Error bars represents SEM. Both models provided very poor fits across the three experiments. Specifically, the models could not even fit well the 4-alternative (a), the 6-alternative (e), and the first choice (i). Not surprisingly, the models also performed poorly for estimating task performance in the 2-alternative (b and f) and the second choice (j) conditions. Specifically, the models underestimated task performance in the 2-alternative and second choice conditions (c, g, and k). Finally, the both models had substantially higher AIC values compared to the summary model (d, h, and l).
