# Supplementary material for: The suboptimality of perceptual decision making with multiple alternatives
Source: Nat Commun. 2020 Jul 31;11:3857. doi: 10.1038/s41467-020-17661-z (PMC7395091; doi:10.1038/s41467-020-17661-z)
Supplement: Supplementary file 2 — Reporting Summary [file 41467_2020_17661_MOESM2_ESM.pdf]

nature research

Corresponding author(s): Jiwon Yeon

Last updated by author(s): May 13, 2020

Reporting Summary

Nature Research wishes to improve the reproducibility of the work that we publish. This form provides structure for consistency and transparency in reporting. For further information on Nature Research policies, see Authors & Referees and the Editorial Policy Checklist.

Statistics

For all statistical analyses, confirm that the following items are present in the figure legend, table legend, main text, or Methods section.

n/a	Confirmed	
☐	☒	The exact sample size (*n*) for each experimental group/condition, given as a discrete number and unit of measurement
☐	☒	A statement on whether measurements were taken from distinct samples or whether the same sample was measured repeatedly
☐	☒	The statistical test(s) used AND whether they are one- or two-sided<br>*Only common tests should be described solely by name; describe more complex techniques in the Methods section.*
☒	☐	A description of all covariates tested
☒	☐	A description of any assumptions or corrections, such as tests of normality and adjustment for multiple comparisons
☐	☒	A full description of the statistical parameters including central tendency (e.g. means) or other basic estimates (e.g. regression coefficient) AND variation (e.g. standard deviation) or associated estimates of uncertainty (e.g. confidence intervals)
☐	☒	For null hypothesis testing, the test statistic (e.g. *F*, *t*, *r*) with confidence intervals, effect sizes, degrees of freedom and *P* value noted<br>*Give P values as exact values whenever suitable.*
☒	☐	For Bayesian analysis, information on the choice of priors and Markov chain Monte Carlo settings
☒	☐	For hierarchical and complex designs, identification of the appropriate level for tests and full reporting of outcomes
☒	☐	Estimates of effect sizes (e.g. Cohen's *d*, Pearson's *r*), indicating how they were calculated

Our web collection on statistics for biologists contains articles on many of the points above.

Software and code

Policy information about availability of computer code

Data collection	MATLAB_R2016a
Data analysis	MATLAB_R2016a, Data analysis codes are available at https://osf.io/d2b9v/files/

For manuscripts utilizing custom algorithms or software that are central to the research but not yet described in published literature, software must be made available to editors/reviewers. We strongly encourage code deposition in a community repository (e.g. GitHub). See the Nature Research guidelines for submitting code & software for further information.

Data

Policy information about availability of data

All manuscripts must include a data availability statement. This statement should provide the following information, where applicable:

- Accession codes, unique identifiers, or web links for publicly available datasets
- A list of figures that have associated raw data
- A description of any restrictions on data availability

All data collected are available at https://osf.io/d2b9v/files/

Field-specific reporting

Please select the one below that is the best fit for your research. If you are not sure, read the appropriate sections before making your selection.

☐ Life sciences ☒ Behavioural & social sciences ☐ Ecological, evolutionary & environmental sciences

For a reference copy of the document with all sections, see nature.com/documents/nr-reporting-summary-flat.pdf

October 2018

Behavioural & social sciences study design

All studies must disclose on these points even when the disclosure is negative.

Study description	The study aims to elucidate what information is represented at the decision stage in perceptual decision making. The study is a quantitative experimental study.
Research sample	A total of 63 subjects participated in the four experiments. Experiment 1 had 32 subjects (15 females, mean age = 20.13, SD = 2.21, range = 18-29), Experiment 2 had 10 subjects (7 females, mean age = 20.5, SD = 3.06, range = 18-28), Experiment 3 had 10 subjects (5 females, mean age = 20.8, SD = 3.55, range = 18-28), and Experiment 4 had 11 subjects (6 females, mean age = 21.45, SD = 2.5, range = 20-28). We used convenience sampling and our final sample is likely to be representative of the college student population but not of the population as a whole. Experiment 1 had a sample size larger than or commensurate with previous studies on human perceptual decision making. In Experiments 2-4, we collected more data per subject and consequently collected fewer subjects as the most important tests were within-subject (as preregistered for Experiments 2 and 3).
Sampling strategy	We used random sampling strategy. We didn't perform sample size calculation. While in the Experiment 1, we recruited a large number of participants, each participant only completed a moderate number of trials (630 trials in total; 420 trials used in the data analysis). For the remaining experiments, we significantly increased the number of trials per participant (3,000 trials/subject) while reducing the total number of participants (n = 10 participants for Experiment 2 and 3, and n = 11 participants for Experiment 4), so that produce reliable modeling for individuals (Steinhauswer et al., 2008).
Data collection	The experiments stimuli were presented on a 21.5-inch iMac monitor in a dark room. The distance between the monitor and the subjects was 60 cm. The stimuli were created and presented at MATLAB_R2016a, using Psychtoolbox 3 (Pelli, 1997). Three undergraduate research assistants helped the data collection but the participants completed the studies alone in the testing booth. The study hypothesis was not blinded to the researcher and all participants were exposed to all experimental conditions.
Timing	Experiment 1 started data collection on March 27th 2017 and completed on April 13th 2017.<br>Experiment 2 started data collection on December 5th 2017 and completed on March 29th 2018.<br>Experiment 3 started data collection on March 6th 2018 and completed on April 14th 2018.<br>Experiment 4 started data collection on May 23rd 2019 and completed on July 15th 2019.
Data exclusions	The 3rd condition (advance warning condition) in Experiment 1 does not fit to the purpose of the current analysis, and thus it was excluded in the data analysis. This exclusion was not pre-registered. Experiments 2-4 did not include a similar condition.
Non-participation	In Experiment 1, three subjects' data were lost due to problems in saving and transferring the data.<br>In Experiment 2, we removed one participant after it was revealed that they were younger than 18 (outside the range of our IRB). Two more subjects failed to complete all 3 sessions.<br>For Experiment 3, one subject voluntarily dropped out after the first session.<br>For Experiment 4, one subject dropped out because the subject turned out to taking neurological medication. Three other subjects decided not to complete the whole 3 sessions of the experiment.
Randomization	We only have one experimental group per each experiment.

Reporting for specific materials, systems and methods

We require information from authors about some types of materials, experimental systems and methods used in many studies. Here, indicate whether each material, system or method listed is relevant to your study. If you are not sure if a list item applies to your research, read the appropriate section before selecting a response.

Materials & experimental systems

n/a	Involved in the study	
☒	☐	Antibodies
☒	☐	Eukaryotic cell lines
☒	☐	Palaeontology
☒	☐	Animals and other organisms
☐	☒	Human research participants
☒	☐	Clinical data

Methods

n/a	Involved in the study	
☒	☐	ChIP-seq
☒	☐	Flow cytometry
☒	☐	MRI-based neuroimaging

Human research participants

Policy information about studies involving human research participants

Population characteristics	See above
Recruitment	Participants contacted us through flyers that we posted over the campus. No bias was present in the recruitment process.

Ethics oversight | The study was approved by the Institutional Review Board of Georgia Institute of Technology

Note that full information on the approval of the study protocol must also be provided in the manuscript.
